# Comparative genomics of *Salmonella enterica* subsp. *diarizonae* serovar 61:k:1,5,(7) reveals lineage-specific host adaptation of ST432

**DOI:** 10.1099/mgen.0.000604

**Published:** 2021-08-02

**Authors:** Laura Uelze, Maria Borowiak, Carlus Deneke, Jennie Fischer, Antje Flieger, Sandra Simon, István Szabó, Simon H. Tausch, Burkhard Malorny

**Affiliations:** ^1^​ Department of Biological Safety, German Federal Institute for Risk Assessment (BfR), Max-Dohrn-Straße 8-10, 10589 Berlin, Germany; ^2^​ Unit for Enteropathogenic Bacteria and Legionella (FG11)/National Reference Centre for Salmonella and Other Bacterial Enteric Pathogens, Robert Koch Institute (RKI), Burgstraße 37, 38855 Wernigerode, Germany

**Keywords:** *Salmonella enterica *subsp. *diarizonae*, sheep, T4SS, type IV secretion system, whole-genome sequencing

## Abstract

Unlike most *Salmonella enterica* subsp. *diarizonae *, which are predominantly associated with cold-blooded animals such as reptiles*,* the serovar IIIb 61:k:1,5,(7) (termed SASd) is regarded as host-adapted to sheep. The bacterium is rarely associated with disease in humans but, nevertheless, SASd isolates are sporadically obtained from human clinical samples. It is unclear whether these transmissions are directly linked to sheep or whether transmissions may, for example, occur through other domestic animals also carrying SASd. For this reason, we utilized whole-genome sequencing to investigate a set of 119 diverse SASd isolates, including sheep-associated and human-associated isolates, as well as isolates obtained from other matrices. We discovered that serovar IIIb 61:k:1,5,(7) is composed of two distinct lineages defined by their sequence types ST432 and ST439. These two lineages are distinguished by a number of genetic features, as well as their prevalence and reservoir. ST432 appears to be the more prevalent sequence type, with the majority of isolates investigated in this study belonging to ST432. In contrast, only a small number of isolates were attributed to ST439. While ST432 isolates were of sheep, human or other origin, all ST439 isolates with source information available, were obtained from human clinical samples. Regarding their genetic features, lineage ST432 shows increased pseudogenization, harbours a *virB/D4* plasmid that encodes a type IV secretion system (T4SS) and does not possess the *iro* gene cluster, which encodes a salmochelin siderophore for iron acquisition. These characteristics likely contribute to the ability of ST432 to persistently colonize the intestines of sheep. Furthermore, we found isolates of the lineage ST432 to be highly clonal, with little variation over the sampling period of almost 20 years. We conclude from the genomic comparisons that SASd underlies a microevolutionary process and that it is specifically lineage ST432 that should be considered as host-adapted to sheep.

## Data Summary

Sequencing data for all *Salmonella enterica* subsp. *diarizonae* serovar 61:k:1,5,(7) isolates analysed in this study have been deposited in the National Center for Biotechnology Information (NCBI) Sequence Read Archive (SRA) under the BioProject accession numbers PRJNA506037, PRJNA637259, PRJEB31846 and PRJNA678834. Supplementary data can be found on Figshare at https://doi.org/10.6084/m9.figshare.14414126.v1.

**Impact Statement**

The majority of *Salmonella enterica* subsp. *diarizonae* serovars are predominantly associated with cold-blooded animals such as reptiles. On the contrary, serovar IIIb 61:k:1,5,(7) (SASd) is generally considered host-adapted to sheep. SASd isolates are also sporadically obtained from human clinical samples. We set out to investigate the links between human-associated and sheep-associated SASd isolates, with the aim to identify matrix-related characteristics. We found that the serovar IIIb 61:k:1,5,(7) consists of two separate lineages (ST432 and ST439), and that lineage ST432 features several host-adaptation traits, which are absent in lineage ST439. Our study has, therefore, uncovered that it is specifically lineage ST432 that should be considered host-adapted to sheep. Furthermore, we found that the investigated ST432 isolates were highly clonal, with little variation over the sampling period of almost 20 years, indicating a stable population. Overall, our study provides an important basis for a deeper understanding of the host-specificity of SASd.

## Introduction

*Salmonella enterica* is a rod-shaped, flagellate, facultative anaerobic, Gram-negative bacterium. The species is divided in six subspecies: *S. enterica* subsp. *enterica* (I), *S. enterica* subsp. *salamae* (II), *S. enterica* subsp. *arizonae* (IIIa), *S. enterica* subsp. *diarizonae* (IIIb), *S. enterica* subsp. *indica* (IV) and *S. enterica* subsp. *houtenae* (VI) [1]. Of the bacteria infecting humans and warm-blooded animals, *S. enterica* subsp. *enterica* is the most prevalent [2]. Other subspecies such as *S. enterica* subsp. *diarizonae* are predominantly thought to be associated with reptiles, and are rarely involved in infections of humans and livestock animals. For example, serovar 61:i:z and serovar 61:c:z35 have been detected in pet snakes [3] and serovar 61:c:1,5,(7) in turtles [4].

Interestingly, there is one serovar that is consistently associated with warm-blooded animals. This *S. enterica* subsp. *diarizonae* serovar 61:k:1,5,(7) (termed SASd) has been found in sheep herds worldwide, with reports from Sweden and Norway [5–7], the US [8], England and Wales [9, 10], Spain [11], Italy [12], Switzerland [13, 14] and Germany [15].

The bacterium colonizes the upper respiratory tract [13] and digestive system of the animals, and can cause diarrhoea [16], abortions [9] and chronic proliferative rhinitis [11, 13]. Although SASd infections in sheep can cause disease, several studies have concluded that the majority of sheep are asymptomatic carriers [6, 17, 18]. Sheep herds are therefore a permanent reservoir of SASd, which may lead to transmission of SASd from sheep to humans. Possible infection routes are direct contact (handling sheep), transmission via contaminated water (through contamination with sheep faeces), or transmission via sheep-derived food products, such as sheep meat, unpasteurized sheep milk or sheep cheese. Overall, however, the zoonotic potential of SASd seems to be low. Only a small number of severe human cases have been described, which mainly affected high-risk groups, such as infants [19], immunocompromised adults [20] and the elderly [21]. No larger outbreak of human infections with SASd has been documented so far and a pseudo-outbreak in France could be traced back to contaminated sheep blood agar [22]. Given the high prevalence of SASd in sheep and the low number of human infections, Swedish authorities have concluded that the risk management of this serovar in sheep can be exempted without any adverse effect on human health [7].

We have previously shown that the genome of a SASd strain isolated from sheep contains genetic features linked to host-adaptation, such as increased pseudogene formation, large-scale genomic rearrangements, a *virB/D4* plasmid and novel genomic islands [23]. We also published the complete genome of a SASd strain obtained from a human clinical sample [24]. In this study, we report a large-scale genome analysis of a diverse set of 119 *S. enterica* subsp. *diarizonae* serovar IIIb 61:k:1,5,(7) isolates from Germany and compare the characteristics of sheep-associated and human-associated isolates.

## Methods

### Strain and database information

We conducted short-read sequencing (Illumina) of 119 *S. enterica* subsp. *diarizonae* serovar 61:k:1,5,(7) isolates from Germany. The dataset includes short-read sequencing data of 16-SA00356 (isolated from sheep) and 14-SA00836-0 (isolated from human urine), two isolates whose complete genomes have been published before [23, 24]. Of the 119 isolates, 55 were derived from sheep (live animal's swabs, organs, aborted foetus, faeces, meat) and 45 isolates were obtained from human clinical samples (urine, stool). The remaining 19 isolates were obtained from various other sources, such as foodstuff (meat, cheese), wild animals (wild boar) and domestic animals (goat, pig, horse, cow, chicken, goose, cat). An overview of all samples, sampling dates and matrices is given in Supplementary File 1. Clinical isolates were provided by the National Reference Centre for Salmonella and Other Bacterial Enteric Pathogens at the Robert Koch Institute (RKI) (Germany). Isolates derived from animals and foodstuffs were provided by the National Reference Laboratory for *Salmonella* at the German Federal Institute for Risk Assessment (BfR) (Germany). Samples originated from 2001 to 2020, with the majority of samples collected within the last 10 years. All strains were randomly selected from the strain collection and have no epidemiological link (neither isolated at the same place, time, animal/human nor food).

In addition, draft assemblies of 202 *S. enterica* subsp. *diarizonae* isolates with the seroformula 61:k:1,5,(7) were extracted from the EnteroBase database [25] and used for comparative purposes. These strains were selected by searching EnteroBase for all strains with a core-genome multilocus sequence typing (cgMLST) hierarchical clustering level of HC2850 equals 1. [In EnteroBase, the HC2850 value corresponds to the separation of subspecies and species, with 1 identifying the subspecies *diarizonae* (https://enterobase.readthedocs.io/en/latest/HierCC_lookup.html)]. The search yielded 1483 strains [27/04/2020]. We downloaded the assemblies of these strains from EnteroBase and determined the serovar with sistr. We then included all strains with the serovar of 61:k:1,5,(7), which were not already encompassed in the data set described above. An overview of the selected strains is given in Supplementary File 2. Of the 202 EnteroBase draft assemblies, 28 isolates were linked to human samples, while 42 isolates were obtained from sheep. Another 28 samples were derived from other animals (pig, cow), foodstuff (dairy, meat) and the environment. No matrix source information was provided for the remaining 104 isolates.

### Whole-genome sequencing and assembly

Bacteria were cultivated on lysogeny broth (LB) agar. A single colony was inoculated in liquid LB and cultivated under shaking conditions (180–220 r.p.m.) at 37 °C for 14–16 h. Genomic DNA was extracted from liquid cultures using a PureLink genomic DNA mini kit (Invitrogen). Sequencing libraries were prepared with the Nextera XT DNA library preparation kit or the Nextera DNA Flex library preparation kit (Illumina), according to the manufacturer’s protocol. Paired-end sequencing was performed on the Illumina MiSeq benchtop sequencer using the MiSeq reagent kit v3 (600 cycle) or on the Illumina NextSeq 500 benchtop sequencer using the NextSeq 500/550 mid output kit v2 or v2.5 (300 cycle). Raw reads were trimmed and *de novo* assembled with the Aquamis pipeline v1.3 (git version is v1.0.0–60-g60e9d09) (https://gitlab.com/bfr_bioinformatics/AQUAMIS), which implements fastp v0.19.5 [26] for trimming and shovill v1.1.0 (https://github.com/tseemann/shovill) for assembly. An overview of the quality-control parameters of the draft assemblies obtained is given in Supplementary File 3.

### Bacterial characterization

All bacterial isolates sequenced for this study were included based on their phenotypical serotyping result according to the White–Kauffmann–Le Minor scheme [27] by slide agglutination with O- and H-antigen specific sera (Sifin Diagnostics). Draft genome assemblies, including those obtained from EnteroBase, were analysed with the BakCharak pipeline v2.0 (git version 1.0.0–77-g5b31a01) (https://gitlab.com/bfr_bioinformatics/bakcharak). The pipeline implements, among other tools, ABRicate v1.0.1 for antimicrobial-resistance and virulence-factor screening (https://github.com/tseemann/abricate), together with the PlasmidFinder database for plasmid detection [28], mlst v2.19.0 (https://github.com/tseemann/mlst), sistr v1.0.2 [29] for *in silico Salmonella* serotyping and Prokka v1.13 [30] for gene annotation. An overview of the results is given in Supplementary File 4. Plasmid contigs were identified with Platon v1.4.0 [31]. Pseudogenes were determined from the short-read draft assemblies of the bacterial isolates with Pseudofinder v0.10 (https://github.com/filip-husnik/pseudo-finder) with standard parameters and length *-l 0.8* (Supplementary File 5). Pseudogene sequences were annotated with Prokka and a presence/absence table of the pseudogenes was computed from the annotated GFF files with Roary v3.13.0 [32]. A customized R script was used to determine those pseudogenes present in ST432 and absent in ST439. Genes of the accessory genome were also identified with Roary v3.13.0 [32] (Supplementary File 6).

Draft genome assemblies were screened with ABRicate v1.0.1 (https://github.com/tseemann/abricate) for the presence/absence of certain genes, such as the type IV secretion system (T4SS) operon and the *iro* gene cluster, with default parameters unless otherwise indicated. The *iro* gene cluster was considered present, if at least four of the five genes (*iroB*, *iroC*, *iroD*, *iroE*, *iroN*) could be detected (Supplementary File 7). The T4SS operon was considered present if at least nine of the eleven genes (*virD2*, *virB1*, *virB2*, *virB4*, *virB5*, *virB6*, *virB8*, *virB9*, *virB10*, *virB11*, *virD4*) could be detected (Supplementary File 8). Screening databases were constructed from nucleotide sequences, as detailed in the respective supplementary files.

### Phylogenetic analysis

cgMLST allele calling was conducted with the chewieSnake pipeline v1.2 [33], which implements chewBBACA v2.0.16 [34]. The cgMLST scheme for *S. enterica* was derived from EnteroBase (https://enterobase.warwick.ac.uk/species/senterica/download_data). SNP calling was conducted with the snippySnake pipeline v1.0 [35], which implements snippy v4.1.0 (https://github.com/tseemann/snippy) for variant calling. Strains 16-SA00356 and 14-SA00836-0 were used as reference sequences for SNP calling. A maximum-likelihood-based phylogenetic tree was inferred with iq-tree v1.6.12 [36] from the core SNPs. Phylogenetic trees were visualized with iTOL v5 [37].

## Results

### Serovar IIIb 61:k:1,5,(7) consists of two separate lineages: ST432 and ST439

**Fig. 1.**
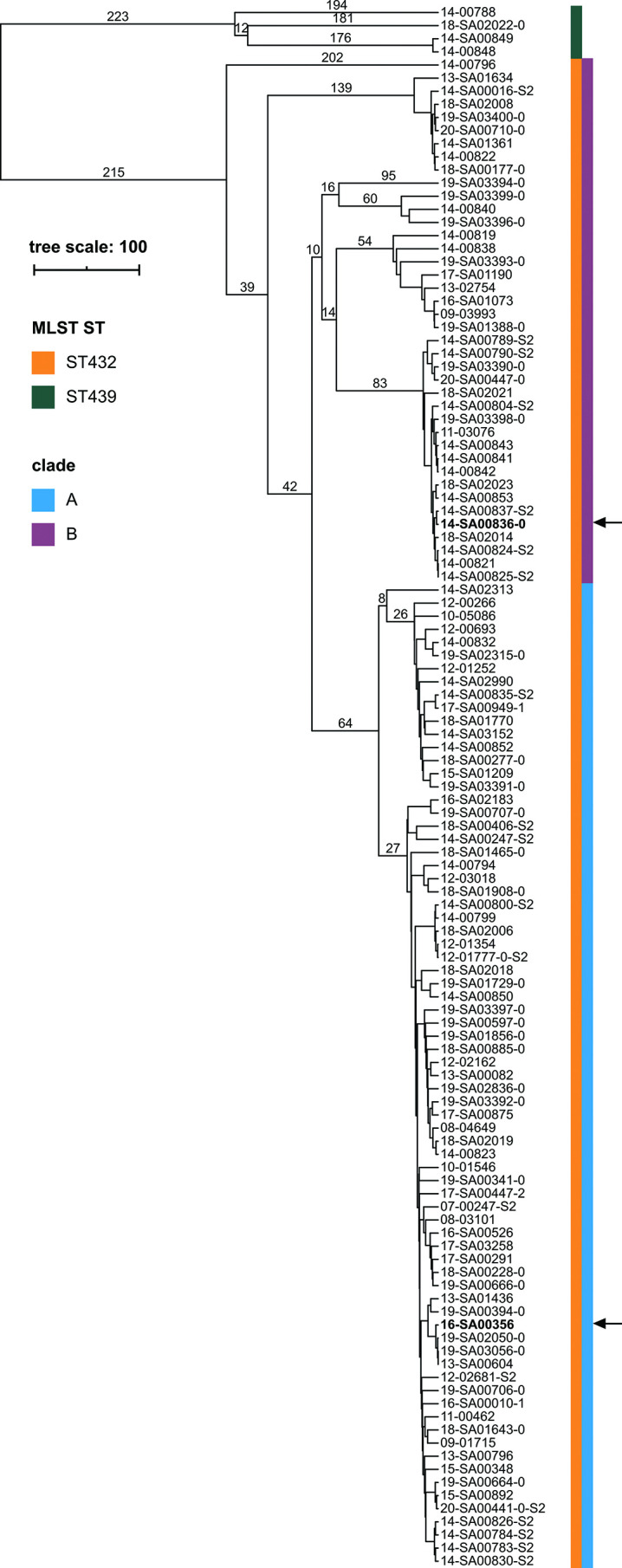
cgMLST minimum-spanning tree of the serovar IIIb 61:k:1,5,(7) isolates. cgMLST allele calling with the draft short-read assemblies was conducted with the chewieSnake pipeline [33], which implements chewBBACA [34]. A minimum spanning tree was computed based on the allele profiles using GrapeTree [66]. The tree was visualized in iTOL [37]. Selected cgMLST allele differences/distances are shown. The scale bar indicates the genomic distances of the sequences in number of allele differences. The left colour bar identifies the 7-gene sequence type (multilocus sequence typing). The right colour bar identifies two separate clades within ST432, termed A and B. Two representative isolates, 16-SA00356 (clade A, isolated from sheep) and 14-SA00836-0 (clade B, isolated from human urine), whose complete genomes have been published before [23, 24], are highlighted in bold and indicated with arrows.

In total, we analysed 321 isolates with the serovar IIIb 61:k:1,5,(7). Of the isolates sequenced for this study (*n*=119), a majority of 115 isolates belonged to ST432, while 4 isolates were assigned to ST439. Of the draft assemblies obtained from EnteroBase (*n*=202), a majority of 197 isolates belonged to ST432, while 5 isolates were assigned to ST439. Therefore, ST432 is the prevailing sequence type in both datasets to a similar extent (97%). The sequence types ST432 and ST439 differ in the *hisD* locus with two SNPs. The two lineages are also clearly discernible by cgMLST (see the minimum spanning tree in Fig. 1). Interestingly, while the 312 isolates of ST432 were both sheep-derived and obtained from human clinical samples (sheep *n*=97, human *n*=65, other=47, unknown *n*=103), the nine ST439 isolates were exclusively attributed to humans (with the exception of one EnteroBase assembly, for which no source information was provided).

### SASd isolates seldomly possess antimicrobial-resistance genes

All SASd short-read draft genome assemblies (*n*=321) were screened for the presence of antimicrobial-resistance genes. The results (Supplementary File 4) show that, with one exception, none of the investigated SASd isolates (both lineages) possess resistance genes to antimicrobials, heavy metals or biocides. The exception is a ST432 isolate (19-SA03391-0), obtained from a human clinical sample, which was found to carry a plasmid-borne *bla*_CMY-2_ resistance gene (plasmid marker *IncI1_1_Alpha*, plasmid size ~98.1 kb), which confers resistance against β-lactam and third-generation cephalosporin antibiotics.

### Lineage ST432 carries a VirB/D4 plasmid, which encodes a T4SS

We detected an IncX4 plasmid in all isolates with ST432, as well as in the majority of the ST432 assemblies obtained from EnteroBase (98%). This plasmid, termed pSASd, was characterized previously from the complete genome sequence of an SASd strain isolated from sheep [23]. The plasmid appears to be highly clonal, as the plasmid sequence of another previously published SASd strain, isolated from human urine [24], was found to be of nearly the same length (42.7 kb±5 bp) with a very high sequence identity of 99.93 % [National Center for Biotechnology Information (NCBI) blastn NZ_CP034075.1 vs NZ_CP054423.1, query cover 100%]. Interestingly, despite the high clonality and wide distribution in ST432, the plasmid-encoded *virB/D4* T4SS was absent from all isolates of lineage ST439 (Supplementary File 8).

The pSASd plasmid is not a typical *Salmonella* virulence plasmid, as it does not contain the *spv* operon [38]. It also does not encompass resistance genes to antimicrobials or heavy metals, but instead encodes several metabolic genes, such as the haemolysin-expression-modulating gene *hha*, DNA-binding protein genes (*hns*), the normally IncN plasmid-related *kikA* (killing in klebsiellas) region and DNA topoisomerase III (*topB*). In addition, it contains toxin–antitoxin stability genes (*relE and stbE*), which likely increase the stability of the plasmid [39].

The largest feature on the pSASd plasmid is a *virB/D4* T4SS, which is encoded by 11 genes [*virD2*, *virB1*, *virB2*, *virB4* (including a *virB3* domain), *virB5*, *virB6*, *virB8*, *virB9*, *virB10*, *virB11* and *virD4*]. The T4SS is a secretion protein complex, which is widely distributed among bacterial species and some archaea [40]. T4SSs contribute to increased virulence and plasmid-borne T4SSs play a role in conjugation [41, 42]. We were interested to see which other *Salmonella* serovar or other species would possess a *virB/D4* T4SS with high sequence similarity to pSASd. Therefore, we performed a blastn search of the T4SS-containing part of the pSASd plasmid [CP054423, range: 20468 (*hha*) – 41604 (*stbE*)] against the NCBI database. We obtained a small number of results (*n*=131) with both high query coverage (≥70 %) and high sequence identity (≥90 %) (Supplementary File 9). The majority of search results were attributed to plasmids of the species *Escherichia coli *, followed by *S. enterica* subsp. *enterica* (serovars Dublin, Enteritidis, Heidelberg, Pullorum, Saintpaul, Typhi and Typhimurium). In addition, the query sequence was also similar to plasmids found in other Gram-negative bacteria of the order *Enterobacterales *. In contrast, no search results of the same high accordance were found for the plasmid backbone (CP054423, range 1–20468), which appears unique to the pSASd plasmid.

We further queried the T4SS operon genes (*virD2*, *virB1*, *virB2*, *virB4*, *virB5*, *virB6*, *virB8*, *virB9*, *virB10*, *virB11*, *virD4*) against our own database of *S. enterica* subsp. *enterica* isolates and against draft assemblies of other subspecies and *Salmonella bongori* obtained from EnteroBase. We found that the prevalence of these genes was unevenly distributed among different serovars (Table 1) (see Supplementary File 8 for full results).

**Table 1.** Prevalence of the T4SS operon in different *Salmonella* spp.

Serovar	Total no. of isolates	Isolates with T4SS (%)
Dublin	34	100
IIIb 61:k:1,5,7 (ST432)	312	99
Heidelberg	5	60
Bovismorbificans	10	10
Newport	22	9
Infantis	253	4
Typhimurium	297	4
Enteritidis	589	2
I 4,[5],12:i:-	313	2
Paratyphi B var. Java	176	2
Derby	91	1
IIIb 61:k:1,5,7 (ST439)	9	0
Agona	89	0
Choleraesuis	37	0
Indiana	41	0
Mbandaka	122	0
Senftenberg	22	0

For example, among the subspecies *enterica* (data source: in-house database), we found that the majority of serovars tested negative for the T4SS operon. A number of serovars, such as Derby (*n*=91), Enteritidis (*n*=589), Paratyphi B var. Java (*n*=176), Typhimurium (*n*=297) and Infantis (*n*=253) occasionally featured the T4SS operon, with 1–4 % of our isolates testing positive. However, all of our Dublin serovar isolates featured the T4SS operon (*n*=34).

The majority of isolates from other *Salmonella* subspecies, as well as *S. bongori* (data source: draft assemblies from EnteroBase) tested negative for the T4SS operon and the operon was only detected in 4 out of 1566 isolates. In contrast, serovar IIIb 61:k:1,5,(7) (ST432) featured a very high prevalence of the T4SS operon, with 98 % of our isolates (*n*=115) and all EnteroBase assemblies (*n*=197) testing positive. However, the serovar IIIb 61:k:1,5,(7), lineage ST439, did not feature the T4SS operon (this study *n*=4, EnteroBase assemblies *n*=5), due to the absence of the pSASd plasmid in this lineage.

### Lineage ST432 features increased pseudogenization

Adaptation to certain eukaryotic hosts often causes bacterial pathogens to undergo pseudogenization of their genome, where previously functional and full-length genes are inactivated, disrupted, eroded and eventually completely removed from the genome [43]. Therefore, it is possible to infer from the number of pseudogenes the level of host adaption [44]. We previously showed that SASd features an increased number of pseudogenes, similar to other host-adapted *S. enterica* subsp. *enterica* serovars, such as serovar Dublin which is bovine-adapted [23]. When comparing the percentage of pseudogenes normalized to the total number of ORFs between the two IIIb 61:k:1,5,(7) lineages, we found ST432 to have a significantly higher share of pseudogenes (5.6 %) compared to ST439 (4.9 %). We also found that compared to other serovars within the subspecies *diarizonae*, ST432 had the highest proportion of pseudogenes (see the full results in Supplementary File 5).

We analysed which genes underwent pseudogenization between ST439 and ST432 and found a set of ten genes, which were pseudogenes in all investigated ST432 and intact genes in ST439 (Table 2). These genes fulfil diverse functions, such as metabolic processes [putrescine degradation (*patA*), lysine utilization (*ldcC*)], binding and transportation of substrates across the membrane [sulfate (*sbp*), α-ketoglutarate (*kgtP*), aldohexuronate (*exuT*)], as well as sensing and signalling of substrate availability [nitrate/nitrite (*narX*), phosphate (*phoH*)]. The gene *ybgQ* is probably involved in fimbrial biogenesis. One gene (*acrD*) encodes a multidrug efflux transporter permease, which in its functional form is involved in the efflux of aminoglycosides. In addition, the *cas3* gene, which is part of the CRISPR-Cas prokaryotic immune system, also underwent pseudogenization in lineage ST432.

**Table 2.** Pseudogenes in ST432, which are functional genes in ST439

Pseudogene name	Locus 16-SA00356 (NZ_CP034074.1)	Locus 14-SA00836-0 (NZ_CP054422.1)	Product	Function
*cas3*	EHF41_RS19780	HTZ89_RS17845	CRISPR-associated helicase	CRISPR-Cas prokaryotic immune system
*sbp*	EHF41_RS22645	HTZ89_RS00815	Sulfate ABC transporter substrate-binding protein	Sulfate binding and transport across the membrane
*phoH*	EHF41_RS14725	HTZ89_RS08210	Phosphate starvation-inducible protein	Unknown
*patA*	EHF41_RS21335	HTZ89_RS19835	Putrescine aminotransferase	Putrescine degradation
*ybgQ*	EHF41_RS18860	HTZ89_RS16970	Fimbrial biogenesis outer membrane usher protein	Probably export and assembly of the putative YbgD fimbrial subunit across the outer membrane
*narX*	EHF41_RS12745	HTZ89_RS10155	Nitrate/nitrite two-component system sensor histidine kinase	Sensor for availability of nitrate/nitrite
*ldcC*	EHF41_RS05800	HTZ89_RS04275	Lysine decarboxylase	Lysine utilization
*kgtP*	EHF41_RS05905	HTZ89_RS16760	α-Ketoglutarate permease	Binding and transport of α-ketoglutarate across the membrane
*exuT*	EHF41_RS21380	HTZ89_RS19880	Hexuronate transporter	Aldohexuronate transport
*acrD*	EHF41_RS06520	HTZ89_RS16160	Multidrug efflux RND transporter permease	Efflux of aminoglycosides

### Lineage ST432 and lineage ST439 differ in their accessory genome

We conducted a pangenome analyses to compare the accessory genomes of the two lineages ST432 and ST439. From the results (Supplementary File 6) two major differences are apparent: (i) ST439 possesses five putative fimbrial genes with similarity to the P/Pap pilus gene cluster of *E. coli* (*papA*, *papB*, *papF*, *papH*, *papK*) [45], which are absent in ST439, and (ii) ST432 lacks five genes involved in salmochelin-mediated iron acquisition (*iroB*, *iroC*, *iroD*, *iroE*, *iroN*) [46], which are present in lineage ST439. In addition, ST432 possesses several other genes [e.g. genes encoding a toxin component of a type I toxin–antitoxin system (*hokC*), an acid shock protein (*asr*), a putative virulence protein (*mkaB*), several nucleases (including *recE*, *ybcO*, *xerS*, *addA*)] that are absent in ST439.

We were intrigued by the absence of the *iro* gene cluster in ST432, as this siderophore has been reported to be present in all *Salmonella* spp. with the exception of *S. bongori* [47]. The *iroB* gene has been found absent in some monophasic Typhimurium (I 4,5,12:i:−), which nevertheless possess the remaining four genes of the *iro* gene cluster [48]. When screening our own database of *S. enterica* subsp. *enterica* isolates for the presence of the *iro* gene cluster, we found that all isolates possessed the five *iro* genes. Equally, when screening ~1540 assemblies of the subspecies *arizonae*, *houtenae* and *salamae*, as well as *diarizonae* (other than IIIb 61:k:1,5,(7)), obtained from EnteroBase, we found all assemblies featured the *iro* gene cluster (Supplementary File 7). Therefore, IIIb 61:k:1,5,(7) lineage ST432 and *S. bongori* are exceptional in that they do not possess the salmochelin siderophore.

### Phylogenetic analysis of ST432 isolates

**Fig. 2.**
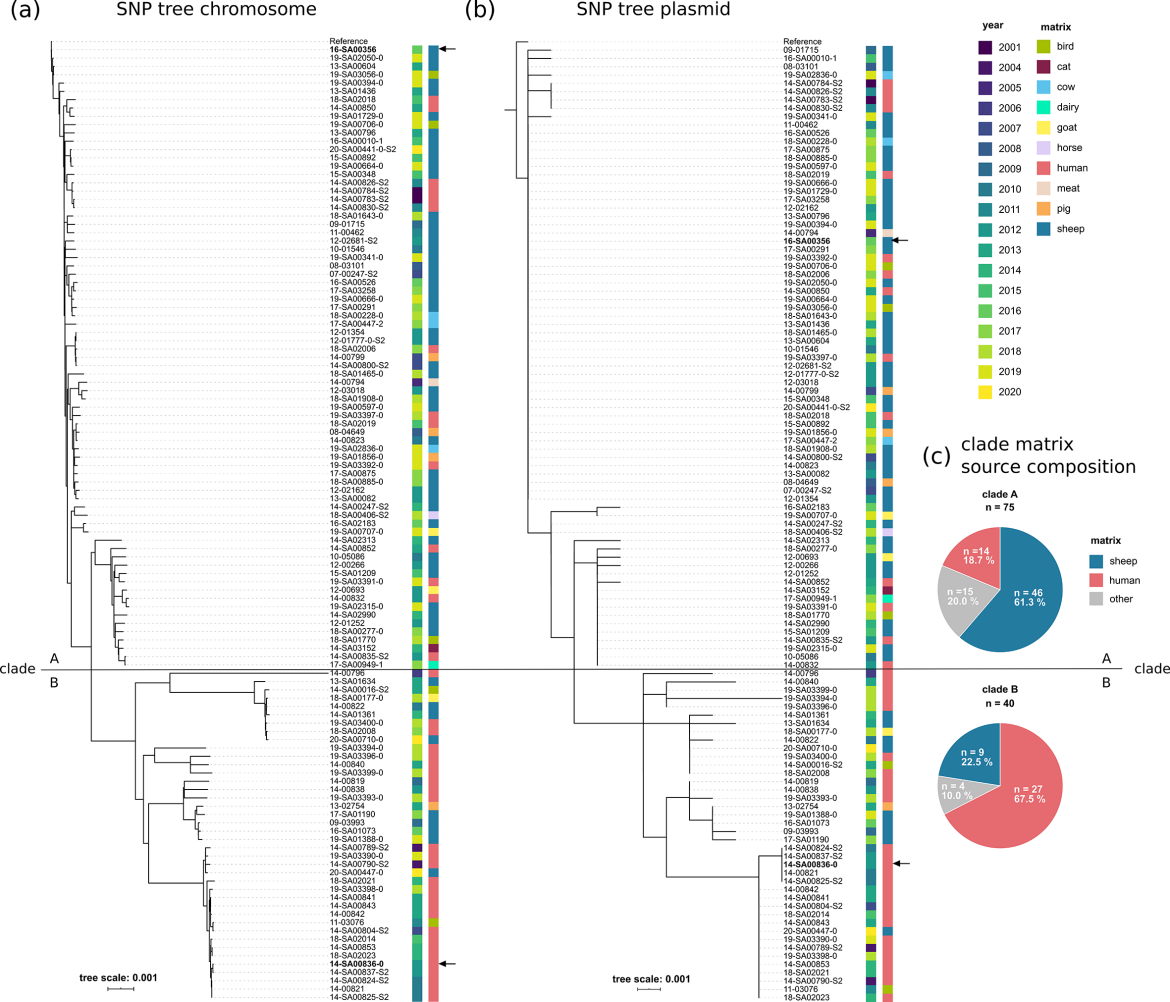
Phylogenetic relationship of the lineage ST432. (**a**) Maximum-likelihood phylogenetic tree based on core SNPs in the bacterial chromosome. The chromosome sequence of 16-SA00356 (NZ_CP034074.1) was used as a reference. (**b**) Maximum-likelihood phylogenetic tree based on core SNPs in the *virB/D4* plasmid (pSASd). The sequence of the plasmid pSE16-SA00356 (NZ_CP034075.1) was used as a reference. Both trees were computed with the snippySnake pipeline [35] with iq-tree [36], visualized in iTOL [37] and manually rooted to the reference. The scale bar indicates the genomic distances of the sequences in substitutions per site. The left colour bars identifies the sampling year. The right colour bars identifies the matrix from which the samples were isolated. Two separate clades, termed A and B, are identified by a horizontal line. Two representative isolates, 16-SA00356 (clade A, isolated from sheep) and 14-SA00836-0 (clade B, isolated from human urine), whose complete genomes have been published before [23, 24], are highlighted in bold and indicated with arrows. (**c**) Pie charts of the matrix source composition for each clade. Matrices other than sheep (blue) and human (red) are grouped into ‘other’ (grey). The total number of isolates for each clade is shown. The respective percentages are indicated in the pie charts.

To infer a phylogeny for isolates with ST432 sequenced in this study (*n*=115), we estimated two maximum-likelihood phylogenetic trees – one based on core SNPs in the bacterial chromosome, the other based on core SNPs in the plasmid sequence (both trees are presented in Fig. 2). Both trees were highly similar in their tree topology. We found that there was an overall low diversity within the isolates, which is shown in the small number of subclusters. Overall, the clustering was independent from isolation year or matrix source. Based on the two main subclusters, we defined two clades, which roughly divide sheep-associated (clade A, *n*=75, 61 % sheep-derived isolates) and human-associated isolates (clade B, *n*=40, 68 % human-derived isolates), with a median number of ~400 SNPs differentiating the two clades. The defined clades are also apparent in a phylogeny estimated from variations in the plasmid sequence (nine SNPs median difference between the two clades), indicating a plasmid with high stability that evolves alongside the genome. For each clade, a representative sample, with a complete genome sequence published earlier, is available. Strain 16-SA00356 [23] isolated from sheep is a representative sample for clade A and strain 14-SA00836-0 [24] obtained from human urine is a representative sample for clade B. Based on these two complete genome sequences, it is possible to estimate the clonality of the two clades. The chromosome sequences of both genomes are of the same length (±320 bp), and have a very high sequence identity of 99.96 % with a query coverage of 99 % (NCBI blastn: NZ_CP034074.1 vs NZ_CP054422.1). Therefore, the two clades are highly similar.

We further attempted to estimate the mutation rate, as well as the divergence time for the emergence/split of lineages ST432 and ST439, using node dating through Bayesian evolutionary analysis with beast2 [49]. However, our analysis failed due to an insufficient temporal signal of the dataset [i.e. the available isolates encompass too few nucleotide substitutions over the sampling period, which caused the calculated mutation rate to drop close to zero (data not shown)].

## Discussion

In this study, we found that *S. enterica* subsp. *diarizonae* serovar IIIb 61:k:1,5,(7) encompasses two distinct lineages, defined by their sequence types ST432 and ST439. The majority of IIIb 61:k:1,5,(7) isolates sequenced to date are of the sequence type ST432, indicating that ST432 is much more prevalent than ST439. All ST439 isolates in our study, for which the source information was available, were isolated from human clinical samples, while ST432 isolates, although commonly isolated from sheep, were also frequently obtained from human clinical samples and occasionally from other sources. We suspect that other studies exploring the prevalence of SASd in sheep have likely examined isolates of ST432 and therefore that most past investigations concern this sequence type. Further research is needed to determine the prevalence, distribution, virulence and pathogenicity of ST439.

Several genetic features distinguish ST432 from ST439, resulting from the adaptation of ST432 to sheep. One defining feature is that ST432 carries a stable and highly clonal *virB/D4* plasmid of ~42.7 kb, which was detected in all isolates sequenced for this study. This plasmid (termed pSASd) encodes a T4SS, which is associated with increased virulence and pathogenicity in several other diverse bacterial pathogens [50]. For example, T4SS is a key virulence factor of *Brucella* spp., such as *Brucella abortus *, that cause abortion in cattle [51].

A similar 32 kb plasmid, also missing the *virB7* domain, was first described in *S. enterica* subsp. *enterica* serovar Heidelberg [52, 53]. A later study investigated the *virB/D4* plasmid in serovar Montevideo [54]. In contrast to our finding that almost all isolates of IIIb 61:k:1,5,(7), lineage ST432, carry this plasmid, these studies found that less than half of the analysed isolates featured the *virB/D4* plasmid (Heidelberg 45 % [53], Montevideo 27 % [54]). Nevertheless, Gokulan *et al*. [52] showed that the *virB/D4* T4SS-containing plasmid contributes to invasion and prolonged survival of the serovar Heidelberg in macrophages and intestinal epithelial cells, due to down-regulating or modulating the immune response of the host. Conversely, a *Caenorhabditis elegans* survival assay failed to demonstrate that serovar Heidelberg strains encoding T4SS genes had a greater pathogenicity, although strains that did not carry any large plasmid and any of the T4SS were statistically less pathogenic [53]. Based on these studies, we hypothesize that the presence of T4SS in lineage ST432 plays a major role in its host-adaptation to sheep. Specifically, the T4SS system may increase uptake and survival of SASd in intestinal epithelial cells, thereby causing the chronic infections of sheep [18]. The high carriage rate of the plasmid in ST432 can be attributed to a stabilizing toxin–antitoxin system on the plasmid, which further highlights the importance of the plasmid for this lineage. Interestingly, we found that the T4SS operon is also highly prevalent in *S. enterica* subsp. *enterica* serovar Dublin (all 34 Dublin isolates encompassed in our *Salmonella* strain collection carry this plasmid). Serovar Dublin, unlike most nontyphoidal *Salmonella* serovars, is host-adapted to cattle, with a wide distribution worldwide. Individual cows can be inapparent carriers of the bacterium throughout their lifetime. This bacterial behaviour is similar to the persistent infection of sheep with SASd and may be associated with the T4SS. Although transmission of SASd between sheep and cows is possible (as some isolates in this study have been isolated from cows), we did not find a very close sequence similarity of the pSASd plasmid to the *virB/D4* T4SS-containing plasmid of serovar Dublin and found that the two plasmids have a different backbone. A recent inter-species transfer of the plasmid is therefore unlikely.

Several accessory genes present in ST432 and absent in ST439 further testify to the host-adaptation of ST432. Importantly, ST432 possesses five putative fimbrial genes with similarity to the P/Pap pilus or fimbrial gene cluster of *E. coli* (*papA*, *papB*, *papF*, *papH*, *papK*) [45]. The P pilus is a fimbrial adhesin that has been studied extensively in *E. coli *. In uropathogenic *E. coli *, the Pap fimbriae form typical rod-like structures and mediate attachment to uroepithelial cells [55]. In *Salmonella *, fimbriae are often required for long-term colonization of the host organism [56]; thus, the five putative fimbrial genes in ST432 likely contribute to the intestinal colonization of sheep.

Another interesting gene present in ST432 and absent in ST439 is *asr*. This gene encodes an acid shock protein and might be involved in the adaptive response to low pH inside the host macrophages [57, 58].

Lineage ST432 is further distinguished by the absence of the *iro* gene cluster. Iron is a limited factor in extraintestinal sites of infection and siderophores such as salmochelins are important virulence factors for pathogens [59, 60]. Although ST432 strains possess other operons encoding siderophores (aerobactin, enterobactin, yersiniabactin), enabling them to acquire iron, the absence of the *iro* gene cluster is intriguing, especially as previous studies have shown that iron acquisition (*iroN*) and metabolism genes (*iroBCDE*) are highly conserved in bovine-associated nontyphoidal *Salmonella* [54].

The fact that the majority of *Salmonella* spp. encode the salmochelin siderophore, but ST432 does not, might point to an interesting relationship between iron acquisition and host adaption. We hypothesize that SASd is exposed to high levels of iron in the rumen and the gut of sheep and, therefore, it does not require the salmochelin siderophore. For example, it was found that in times of food scarcity, sheep start to graze down closer to the ground, causing them to ingest large quantities of iron-rich soil [61], which may result in an abundance of iron [62]. However, another explanation is certainly possible and further research is needed to investigate why ST432 is characterized by the absence of the *iro* gene cluster, which is highly conserved in all other *Salmonella* spp. (with the exception of *S. bongori *).

The absence of antimicrobial-resistance genes in ST432 (with one exception, no resistance genes were detected in either ST432 or ST439) is likely a consequence of the low intensity with which sheep are farmed. Compared to high-intensity farmed animals such as chicken or pigs, antibiotic usage in sheep tends to be lower [63]. As a consequence, SASd is exposed to little evolutionary pressure, which otherwise would lead to the development of antimicrobial resistances [64].

As we described previously [23], SASd features an increased number of pseudogenes. Pseudogenization is an indicator of host adaptation, as genes that are no longer required are inactivated through disruption of the genetic sequence. In this study, we found that lineage ST432 has a markedly higher share of pseudogenes (5.6 %) than ST439 (4.9 %). Interestingly, one of the pseudogenes in ST432 is *cas3*. A recent publication [65] showed that *Δcas3* knock-out *S. enterica* subsp. *enterica* serovar Enteritidis mutants had decreased virulence, thereby increasing the survival of infected host cells. In addition, the *Δcas3* mutant featured downregulated biofilm-related genes and *Salmonella* pathogenicity island 1 (SPI-1) genes related to the type III secretion system (T3SS). The fact that *cas3* is naturally inactivated in lineage ST432 may be yet another consequence of its host-adaption to sheep. For example, it is possible that a decreased biofilm formation may be due to a decreased need to persist in an environmental reservoir. However, a decreased pathogenicity and virulence might allow long-term persistent infections, as host-adapted organisms benefit from a decreased mortality rate of their host organism. We did not test the ability of SASd ST432 to form biofilms *in vivo* and subsequent studies are needed to further explore the biofilm formation capacity of this organism.

Although we found that ST432 isolates separated in two clades, with one clade featuring more human-associated samples and one clade containing more sheep-associated samples (albeit only at a level of 60 %), the low overall diversity and high clonality of SASd, due to a very low mutation rate over time, seems to indicate that there are no divergent subpopulations of SASd. Furthermore, the fact that a total of 19 SASd isolates were obtained from neither human nor sheep, but instead from foodstuff, other domestic animals and pet animals, demonstrates that SASd can be transmitted to a variety of species and environments. Therefore, it can be concluded that although sheep may be the main reservoir of SASd, human infections of SASd do not need to directly be linked to sheep, but may be caused by other domestic and companion animals, as well as the environment and foodstuff.

### Conclusion

The findings in this study reveal that *S. enterica* subsp. *diarizonae* serovar 61:k:1,5,(7), termed SASd and considered host-adapted to sheep, is composed of two separate lineages. These two lineages are identified by their 7-gene sequence type: ST432 and ST439. The two lineages differ in the following characteristics: (i) ST432 carries a plasmid-borne T4SS, which likely contributes to persistent infections of the host organism (i.e. sheep) and which is absent in ST439; (ii) ST432 has a higher proportion of pseudogenes (5.6 %), compared to ST439 (4.9 %), as a result of continued genetic adaptation to the host environment; and (iii) ST432 lacks the *iro* gene cluster, consisting of five genes involved in salmochelin-mediated iron acquisition, which is present in ST439, as well as in most other *Salmonella* spp. Combined with the finding that ST432 is often isolated from sheep, while the nine ST439 isolates involved in this study were obtained from humans, we conclude that it is specifically lineage ST432 that should be considered as host-adapted to sheep. Therefore, we suggest that the term SASd should be reserved for IIIb serovar 61:k:1,5,(7) lineage ST432, and that future prevalence studies of SASd in sheep should include information about the sequence type of the isolates, where possible. Due to the small number of isolates with ST439 investigated in this study, further research is needed to identify the reservoir, prevalence and pathogenicity of the ST439 lineage.

## Supplementary Data

Supplementary material 2

Supplementary material 2

Supplementary material 2

Supplementary material 2
